# Connective tissue growth factor promotes articular damage by increased osteoclastogenesis in patients with rheumatoid arthritis

**DOI:** 10.1186/ar2863

**Published:** 2009-11-18

**Authors:** Kazuhisa Nozawa, Maki Fujishiro, Mikiko Kawasaki, Hiroshi Kaneko, Kazuhisa Iwabuchi, Mitsuaki Yanagida, Fujihiko Suzuki, Keiji Miyazawa, Yoshinari Takasaki, Hideoki Ogawa, Kenji Takamori, Iwao Sekigawa

**Affiliations:** 1 Institute for Environment and Gender Specific Medicine, Juntendo University Graduate School of Medicine, Chiba, 2-1-1 Tomioka, Urayasu, Chiba, 279-0021, Japan; 2 Department of Internal Medicine and Rheumatology, Juntendo University Urayasu Hospital, 2-1-1 Tomioka, Urayasu, Chiba, 279-0021, Japan; 3 Department of Pathology, Juntendo University Urayasu Hospital, 2-1-1 Tomioka, Urayasu, Chiba, 279-0021, Japan; 4 Central Research Laboratories, Kissei Pharmaceutical Co Ltd, 4365-1 Hotakakashiwabara, Azumino, Nagano, 399-8304, Japan; 5 Department of Rheumatology, School of Medicine, Juntendo University, 2-1-1 Hongo, Bunkyo, Tokyo, 113-8421, Japan

## Abstract

**Introduction:**

A protein analysis using a mass spectrometry indicated that there are serum proteins showing significant quantitative changes after the administration of infliximab. Among them, connective tissue growth factor (CTGF) seems to be related to the pathogenesis of rheumatoid arthritis (RA). Therefore, this study was conducted to investigate how CTGF is associated with the disease progression of RA.

**Methods:**

Serum samples were collected from RA patients in active or inactive disease stages, and before or after treatments with infliximab. CTGF production was evaluated by ELISA, RT-PCR, indirect immunofluorescence microscopy, and immunoblotting. Osteoclastogenesis was evaluated using tartrate-resistant acid phosphatase (TRAP) staining, a bone resorption assay and osteoclasts specific catalytic enzymes productions.

**Results:**

The serum concentrations of CTGF in RA were greater than in normal healthy controls and disease controls. Interestingly, those were significantly higher in active RA patients compared to inactive RA patients. Furthermore, the CTGF levels significantly were decreased by infliximab concomitant with the disease amelioration. In addition, tumour necrosis factor (TNF)α can induce the CTGF production from synovial fibroblasts even though TNFα can oppositely inhibit the production of CTGF from chondrocytes. CTGF promoted the induction of the quantitative and qualitative activities of osteoclasts in combination with M-CSF and receptor activator of NF-κB ligand (RANKL). In addition, we newly found integrin αVβ3 on the osteoclasts as a CTGF receptor.

**Conclusions:**

These results indicate that aberrant CTGF production induced by TNFα plays a central role for the abnormal osteoclastic activation in RA patients. Restoration of aberrant CTGF production may contribute to the inhibition of articular destruction in infliximab treatment.

## Introduction

Rheumatoid arthritis (RA) is a chronic inflammatory disorder that ultimately leads to the destruction of the joint architecture. Although the precise pathogenic mechanisms leading to the development of RA are not fully understood, proinflammatory cytokines, such as tumor necrosis factor-α (TNF-α), interleukin (IL)-1 and IL-6 play pivotal roles in the induction of RA [1-4]. Especially, TNF-α is considered to play a central role in bone destruction because TNF-α mediates an abnormal activation of osteoclasts through either the direct or indirect mechanisms in RA [2,3]. The use of TNF-α blockade reagents has been shown to have a significant impact on the therapy of RA and the success of this therapy has led to trials in other chronic inflammatory diseases such as Behcet's disease [5-8]. Infliximab is chimeric IgG1 anti-TNF-α antibody containing the antigen-binding region of a mouse antibody and the constant region of human antibody [9]. The antibody binds soluble and membrane bound TNF-α, thereby impairing binding to its receptor. Although anti-TNF-α blocking reagents possess a beneficial effect for RA therapy especially for prevention of articular destruction, the precise mechanism of the disease's amelioration has not been clarified because TNF-α has multiple functions and it is involved in many inflammatory pathways and it also regulates various physiological phenomena in RA patients [7,8].

A previous study has shown the changes in the profiles of serum protein biomarkers in infliximab-treated RA patients. It was achieved by a novel approach to proteomic research using a specially developed serum/plasma protein separation device (hollow-fiber-membrane-based device; HFRD, Toray Industry, Tokyo, Japan) and a linked two-dimensional liquid chromatography system (2D LC-MS/MS) [10]. Various proteins (approximately 20 kinds of proteins) revealed great changes in their expression after the infliximab treatment using this analytical system, however, many proteins among them were cellular constitutive proteins. These were thought to be released into sera from cells destroyed by anti-TNF-α antibodies because the antibodies are known to mediate the killing of cells expressing TNF-α on the surface [9]. Among these proteins listed in the previous study [10], connective tissue growth factor (CTGF) appeared to be a potent strong biomarker in the infliximab-treated RA patients. CTGF was discovered due to the cross-reactivity of a platelet derived growth factor (PDGF) antiserum with a single polypeptide with a molecular weight of 38 kDa secreted by cultured human vein endothelial cells (HUVEC), and its cDNA was isolated from a HUVEC cDNA expression library with anti-PDGF and shown to encode a 349-amino acid protein [11]. CTGF is a member of the CCN protein family (including Cyr61 (CCN1), CTGF (CCN2) and Nov) and believed to be a downstream mediator of transforming growth factor (TGF)-α action [12]. Although a number of cell surface molecules have been nominated as candidates currently for its specific receptors, they have not been defined to date. CTGF is a bioactive cytokine, therefore, it is considered not to be derived from these destroyed cells. Furthermore, it has been shown that CTGF is associated with several biological functions such as fibrosis, tumorgenesis, angiogenesis, and endochondral ossification, and it has been proposed that CTGF produced by chondrocytes might maintain a homeostasis of cartilage tissue by autocrine system [13,14]. Articular tissue consists of not only chondrocytes but also various kinds of cells such as synovial fibroblasts or osteoclasts. Especially, fibroblasts of inflamed synovial tissue and osteoclasts are thought to be the main effecter cells for the development of bone destruction in RA. However, precise functions of CTGF on these articular cells have not been elucidated so far.

Based on these findings, the contribution of CTGF for RA pathogenesis was investigated in the current study. Here, we report that aberrant CTGF production mediated by TNF-α can induce massive osteoclastogenesis and disturbance on homeostasis of cartilage resulting in bone and cartilage tissue damage in RA. Furthermore, we report here that phosphorylated extracellular signal-regulated kinase (ERK) 1/2 was recruited by CTGF stimulation on activation of the signal transduction pathway associated with integrin αVβ3 and contributed to focal adhesion kinase (FAK) activation on the osteoclasts. These data indicate that we found integrin αVβ3 as a CTGF receptor on the osteoclasts. We insist that CTGF is a potentially novel effecter molecule for RA pathogenesis and our data could help better understanding for elucidation of the protective mechanisms for bone destruction associated with the efficacy of infliximab treatment. The blockade of the anti-CTGF/integrin αVβ3 pathway might become a new useful strategy for the treatment of RA.

## Materials and methods

### Patients and samples

All patients with RA and systemic lupus erythematosus (SLE) fulfilled the American College of Rheumatology (ACR) criteria [15]. All patients with Sjögren's syndrome (SS) also fulfilled the American-European Consensus Criteria (AECC) [16]. Serum samples were obtained from 39 patients with RA, 11 patients with SLE, 4 patients with primary SS and 50 normal age- and gender-nearly matched healthy volunteers. The synovial tissue samples were obtained from two patients with RA and osteoarthropathy (OA) as disease controls during a surgical operation for knee joints arthropathy. The patients with RA were further categorized as an active RA group (n = 20) and inactive RA group (n = 19) depending on the elevated serum C-reactive protein (CRP) level (normal range < 0.3 mg/dl). The active RA group includes the patients who had received infliximab treatment (n = 10). The precise clinical profiles of these patients had been described in a previous report and all these patients had shown disease amelioration by the infliximab treatment [10]. All patients provided their informed consent to participate in this study and the study was approved by the local ethics committee.

### Materials and cell lines

A human synovial fibroblasts cell line, MH7A (Riken Cell Bank, Ibaraki, Japan), isolated from the knee joint of RA, was provided by Dr. Miyazawa [17]. A human chondrogenic cell line OUMS-27 was purchased from Health Science Research Resource Bank (Tokyo, Japan) [18]. MH7A cells and OUMS-27 cells were cultured in Dulbecco's modified Eagle's medium (DMEM: Sigma; St. Louis, MO, USA) containing 10% fetal bovine serum (FBS) under standard conditions. MH7A cells and OUMS-27 cells were stimulated with or without recombinant TNF-α (20 ng/ml in MH7A cells, 50 ng/ml in OUMS-27 cells, respectively) (R&D System; Minneapolis, MN, USA) in appropriate time (6 and 24 or 48 hours) and used for the subsequent experiments. Treatment with 1 μg/ml of infliximab was used to inhibit effects of TNF-α in vitro in the experiments.

### ELISA for human CTGF

The serum level of CTGF in human sera was evaluated by a sandwich ELISA system using two different anti-human CTGF antibodies; monoclonal anti-human CTGF antibody (R&D System, Cat#MAB660) and biotinated anti-human CTGF antibody (R&D System, Cat#BAF660). To reduce non-specific reactions and gain a higher sensitivity, high molecular weight proteins contained in the sera were removed by Multiple Affinity Removal Spin Cartridge Reagent Kit (Agilent Technologies, Santa Clara, CA, USA) and used for the serum samples. Monoclonal anti-CTGF antibodies (R&D Systems) were diluted in phosphate-buffered saline (PBS) to a final concentration of 10 μg/ml and then coated on Optiplate-96F microtiter plates (PerkinElmer, Waltham, Massachusetts, USA). After a blocking step, the serum samples were diluted 1:150 in PBS and then incubated in the antibody-coated wells at 4°C overnight. Biotinylated anti-CTGF antibody (R&D systems) was used at 2 μg/ml dilution for the detection and then beta galactosidase conjugated streptavidin (Rockland Immunochemical for Research, Gilbertsville, PA, USA) was added. 4-methylumbelliferyl-β-D-galactoside (Research Organics, Cleveland, OH, USA) was used as the detection reagent. Recombinant human CTGF protein (Biovender Laboratory Medicine Inc, Modrice, Czech Republic) was used as a standard for the quantitation. Each sample was analyzed in triplicate and the average optical density (OD) at 460 nm with an appropriate development time was used for the data analysis.

### Total RNA extraction and real-time RT-PCR

Total RNA was extracted from the MH7A cells, OUMS-27 cells and osteclasts using the Rneasy Mini Kit (QIAGEN, Hilden, Germany) according to the manufacturer's instructions. Strands of cDNA were synthesized using a PrimeScript RT reagent kit (Takara, Shiga, Japan) with 0.5 μg total RNA. Real-time RT-PCR using SYBR Premix Ex Taq Perfect real time (Takara) was used for the quantitation of CTGF mRNA. The primers for human CTGF [GenBank:NP_001892] were designated as 5' CTTGCGAAGCTGACCTGGAA-3' (forward) and 5'-AGCTCAAACTTGATAGGCTTGGAGA-3' (reverse), β-actin primers for control primers as 5'-TGGCACCCAGCACAATGAA-3' (forward) and 5'-CTAAGTCATAGTCCGCCTAGAAGCA-3' (reverse), Cathepsin-K primers as 5'-AGCTGCAATAGCATAATCTGAACC (forward) and 3-CGTTGTTCTTATTTCGAGCCATGA (reverse) and matrix metalloproteinase (MMP)-9 primers as 5-ACCTCGAACTTTGACAGCGACA (forward) and 3-GATGCCATTCACGTCGTCCTTA (reverse). Quantitative real-time RT-PCR was performed in 20 μl volume with 500 ng cDNA in SYBR Premix Ex Taq Kit (Takara). The amplification cycles consisted of 95°C for five seconds as first steps (one cycle), 95°C for five seconds and 60°C for 30 seconds for CTGF as second steps (45 cycles), 95°C for five seconds and 60°C for 30 seconds and 95°C for 15 seconds as third steps (one cycle) according to protocol described in the manufacturer's instructions (Takara). To determine the quantitative expression levels of the transcripts, samples loading was monitored and normalized by the expression of β-actin transcripts.

### Osteoclasts differentiation

Peripheral blood monocytes (PBMC) from healthy donors were collected using Ficoll-gradient centrifugation (Ficoll-Paque PLUS, GE Healthcare, Chalfont St Giles UK). The PBMC were purified into a CD14+ population using anti-CD14 MACS microbeads (Miletenyi Biotec, Auburn, CA, USA) according to the protocol supplied by the manufacturer. A flow cytometory analysis using phycoerythrin (PE)-conjugated mouse anti-CD14 mAb (Miletenyi Biotec) showed that purity of the CD14+ monocytes was more than 98% in each experiment. The purified CD14+ monocytes (5 × 10^4 ^cells/well) were cultured in 96 wells in alpha minimum essential medium (αMEM, Invitrogen, Grand Island, NY, USA) with 10% FBS and incubated with M-CSF (25 ng/ml) and soluble RANKL (sRANKL; 40 ng/ml) (Millipore, Billerica, MA, USA) with or without bioactive recombinant CTGF (1 μg/ml) (Biovender Laboratory Medicine Inc, Brno, Czech Republic). The medium was replaced with fresh medium three days later and the cells were stained for tartrate-resistant acid phosphatase (TRAP) expression using a commercial kit (Cell Garage, Tokyo, Japan) after incubation for seven days. The number of TRAP positive multinucleated cells (MNC) in three randomly selected fields examined at 100× magnification of the total number of TRAP-positive MNC per well were counted as osteoclasts under light microscopy. For immunoblotting and immunoprecipitation analysis, osteoclasts were initially differentiated by M-CSF (25 ng/ml) and sRANKL (40 ng/ml) without CTGF for seven days. Then, recombinant CTGF (10 or 50 ng/ml) was added into the cultures and incubated at 5, 15, 60, and 120 minutes in the presence or absence of anti-CTGF antibody (1 μg/ml). The cells were washed and collected for making cell extracts for subsequent immunoblotting and immunoprecipitation assays.

### Immunoblotting and immunoprecipitation

MH7A and OUMS-27 cells were centrifuged at 200 × g for 30 min. Cell pellets (1 × 10^7 ^cells/well) were then resuspended directly in lysis buffer containing 150 mM NaCl, 1 mM MgCl_2_·6H_2_O, 80 mM Tris-HCl, 0.1% NP-40 and Complete Protease Inhibitor cocktail (Roche, Mannheim, Germany). The protein concentrations in the lysates were determined using the Protein DC Assay Kit (Bio-Rad, Hercules, CA, USA) to ensure equal loading of proteins in each SDS-PAGE lane. After determining the protein concentration, lysates were mixed with an equal volume of 2 × gel sample buffer containing 6% sodium dodecyl sulfate, 20% glycerol, 10% β-mercaptoethanol, 0.02% bromophenol blue and Complete Protease Inhibitor cocktail. Lysates were stored at -80°C until use. The equivalent of 10 μg total lysate protein was loaded onto each lane of 12.5% SDS-PAGE gels, separated by electrophoresis and then transferred to nitrocellulose membranes using a Semi-Dry Trans-Blot apparatus (Bio-Rad). After blocking, immunoblotting was performed using polyclonal goat anti-human CTGF (L-20) antibody (Santa Cruz Biotechnology, Santa Cruz, CA, USA) at 1:2,000 and monoclonal mouse anti-human β actin antibody (Sigma) at 1:500 dilution. The detection of bound antibodies was achieved using horseradish peroxidase-conjugated anti-goat IgG antibody and anti-mouse IgG antibody (Dako, Glostrup, Denmark) used at 1:5,000 and 1:2,500 dilution respectively, in combination with enhanced chemiluminescence (Super Signal West Pico, PIERCE Products, Rockford, IL, USA). Cell extracts of the osteoclasts stimulated with or without recombinant CTGF were prepared with the same condition for subsequent immunoblotting and immunoprecipitation assays. For immunoprecipitation, 5 μg of mouse anti-human integrin αVβ3 antibody (Chemicon International, Temecula, CA, USA) were incubated at 4°C overnight with protein G-conjugated agarose beads. Samples were washed and resuspended in 2 × gel sample buffer and boiled prior to immunoblotting. The immunoprecipitation samples or the cell extract were further subjected to SDS-PAGE and electro-transferred to the membranes. After blocking, primary antibodies; mouse anti-human integrin αVβ3 antibody (Chemicon International) at 1:2,000 dilution, mouse anti-human ERK1/ERK2 antibody (Abcam, Cambridge, MA, USA) at 1:2,000 dilution, and mouse anti-human phospholilated ERK1/ERK2 antibody (Abcam) at 1:10,000 dilution were applied for the blots at 37°C, 120 minutes. For immunoblotting assay, osteoclasts were stimulated with recombinant CTGF (10 and 50 ng/ml) at 60 minutes, then, the extracts were subjected to SDS-PAGE and electro-transferred to the membranes. The primary antibodies; mouse anti-human β actin antibody (Sigma) at 1:500 dilution, rabbit anti-human focal adhesion kinase (FAK) antibody (Santa Cruz Biotechnology, Santa Cruz, CA, USA) at 1:200 dilution, rabbit anti-human phospholilated FAK at 1:200 dilution were incubated with the blots at 37°C, 120 minutes. The detection of bound antibodies was achieved using horseradish peroxidase-conjugated anti-goat IgG antibody, anti-mouse IgG antibody, and anti-rabbit IgG antibodies (Dako, Denmark) used at 1:5,000 dilution.

### Immunohistochemistry analysis

A histochemical analysis with indirect immunofluorescence microscopy was performed. Briefly, serial paraffin sections derived from surgical samples were deparaffinized, rehydrated and washed with PBS as previously reported [19]. Double staining for CTGF and F4/80, which is widely used as a specific marker for macrophage, was performed. The samples were incubated with 10% bovine serum albumin (Sigma) for 60 minutes to eliminate nonspecific binding, and then incubated with goat anti-human CTGF (L-20) antibody (Santa Cruz Biotechnology, Santa Cruz, CA, USA) and rat anti-human F4/80 antibody (Abcam) for 60 minutes diluted 1:50 in PBS. After washing, the bound antibodies were labeled with Alexa468 (Invitrogen Corporation, Carlsbad, CA, USA) conjugated anti-goat IgG antibody (Invitrogen Corporation, Carlsbad, CA, USA) and Alexa594 conjugated anti-rat IgG antibody (Molecular Probe) for detection of fluorescence images. The sections were counterstained by the nuclear stain 40,6-diamidino-2-phenylindole (DAPI) (Vector Laboratories, Burlingame, CA, USA). The other sections were also stained with hematoxylin/eosin (HE).

#### Resorption assay

Purified CD14+ monocytes were seeded onto plates coated with calcium phosphate thin films (Biocoat Osteologic, BD Biosciences, San Jose, CA, USA) and were incubated with sRANKL (40 ng/ml) + M-CSF (25 ng/ml) in combination with or without CTGF (1 μg/ml) for seven days. The cells were then lysed in bleach solution (6% NaOCl, 5.2% NaCl). The resorption lacunae were examined under light microscopy.

### Statistical analysis

The experimental data were compared using un-pared Student's t-test with *P *values < 0.05 considered to be statistically significant.

## Results

### Increased serum levels of CTGF in patients with RA

**Figure 1:**
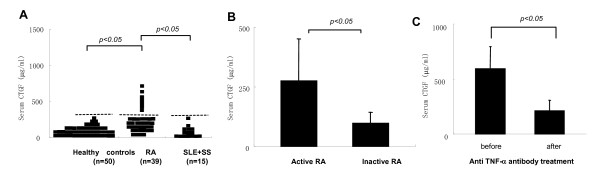
The serum levels of connective tissue growth factor in patients with rheumatoid arthritis. The serum concentration of connective tissue growth factor (CTGF) in patients with RA (n = 39), SLE and SS (n = 15) and normal healthy controls (n = 50) were measured using a sandwich ELISA (Figure 1A). Dotted line (Figure 1A) indicates the cut off point designed as the mean OD + 3 SD for normal sera. Value above the cut off point was defined as an elevated serum CTGF concentration. Comparison of the serum concentrations of CTGF between active (n = 20) and inactive (n = 19) RA patient was shown (Figure 1B). Comparison of the serum concentrations of CTGF between before and after Infliximab treatment was also shown (Figure 1C). The sera treated with infliximab (n = 10) were collected at post 24 hrs after the first administration. Bars in 1B and 1C indicate the SD.

Figure 1A shows the serum levels of CTGF in the patients with RA, disease controls (SLE and SS) and normal controls. The serum levels of CTGF in RA patients were significantly greater in comparison to normal controls or disease controls (*P *< 0.05). There were no significant differences in the serum CTGF concentrations between disease controls and normal controls. When cut off value (dotted line) was defined as the mean value of normal controls + three standard deviations [20], 25.8% of RA patients (eight in 39 patients) showed elevation of serum CTGF level. In contrast, none of the disease and normal controls showed any elevation of CTGF levels in their sera.

### Correlation between CTGF and diseases activity

Serum CTGF levels were compared between the patients having high and low RA disease activities (Figure 1B) and the effect of infliximab on the serum levels of CTGF was examined in RA patients (Figure 1C). We could not fully obtain information of articular manifestations with these patients, therefore, the patients were provisionally divided into two groups, active/inactive RA, depending upon the serum level of CRP (active RA; CRP > 0.3 mg/ml, inactive RA; CRP < 0.3 mg/ml). Other comparative parameters such as erythrocyte sedimentation rate (ESR), white blood cell (WBC), and matrix metalloprotease-3 (MMP-3) were also significantly elevated in the active RA group (Table 1). As shown in Figure 1B, the levels of serum CTGF were significantly elevated in active RA compared with inactive RA. Interestingly, the frequency of sera with elevated CTGF levels designed as above the cut off points was significantly greater in the active RA group than the inactive RA group (7/20 vs 1/19, *P *< 0.05) (Table 1). Figure 1C shows a reduction of serum CTGF levels in response to infliximab treatment. The statistical reduction of serum CTGF levels was observed after infliximab treatement (*P *< 0.05) (Figure 1C). These data suggest that serum levels of CTGF correlate with the disease activity and concern a pathogenesis of RA.

**Table 1:** Patient profile with active/inactive rheumatoid arthritis

	Active RA (n = 20)	Inactive RA (n = 19)	*P *value
CRP (mg/ml)	3.38 ± 2.83	< 0.3	*P *< 0.01
ESR (mm/h)	69.9 ± 29.7	19.5 ± 13.2	*P *< 0.05
WBC (cells/ml^3^)	8235 ± 3200	5470 ± 552	*P *< 0.05
MMP-3 (ng/ml)	402.0 ± 307.9	103.1 ± 73.3	*P *< 0.05
Frequency of CTGF elevated sera	35.0% (7/20)	5.3% (1/19)	*P *< 0.05

CTGF = connective tissue growth factor; RA = rheumatoid arthritis; CRP = C reactive protein; ESR = erythrocyte sedimentation rate; WBC = white blood cell; MMP = matrix metalloproteinase.

### Effects of TNF-α for the production of CTGF from synovial fibroblasts or chondrocytes

**Figure 2:**
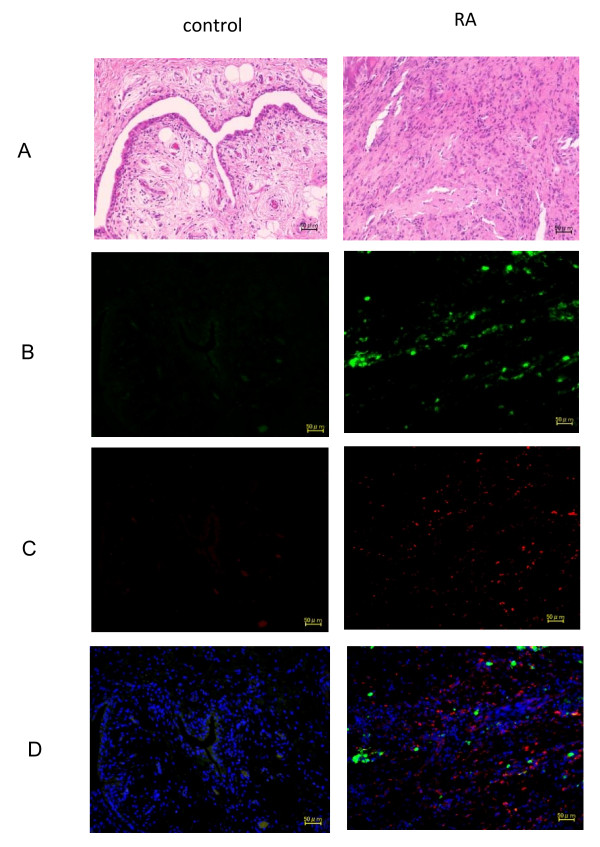
Connective tissue growth factor expression in synovial tissue of patients with rheumatoid arthritis. The representative results of HE staining (Figure 2A), immunofluorescence anti-CTGF antibody staining (Figure 2B; green), and anti-F4/80 antibody staining (Figure 2C; red) are shown using surgical samples from RA and OA patients. The samples were counterstained by DAPI (blue) for nuclear staining and merge images are shown (Figure 2D). A strong expression of CTGF and F4/80 was observed in the samples of RA compared to OA and the CTGF expression cells were not overlapped with F/40 expression cells indicating that CTGF is upregulated in synovial fibroblasts rather than macrophages.

To investigate the CTGF contribution for pathogenesis of RA, the CTGF expression was evaluated by immunohistochemical analysis in synovial tissues of surgical samples from the knee joint of RA patients and OA patients as a disease control. An inflamed synovial tissue was recognized in samples from RA compared to OA in HE staining (Figure 2A). A strong expression of CTGF was observed in inflamed synovial tissue with RA, and none or very weak expression was recognized in samples with OA (Figure 2B). Inflamed synovial tissue of RA generally consists of fibroblasts and lineage of hemapoietic cells such as macrophage, neutrophils, and lymphocytes. To investigate a more specific production site of CTGF, the samples were also stained by anti-F4/80 antibody, which is generally used for a specific marker of macrophages. As expected, a significant infiltration of macrophages was observed in RA compared to OA (Figure 2C). Double staining using anti-CTGF antibody and anti-F4/80 antibody showed that expression of CTGF and F4/80 was not overlapped (Figure 2D), suggesting that these molecules were produced at different sites. Because it has been reported that CTGF was not generally expressed in hemopoietic lineage cells [14], we considered that CTGF was mainly produced at synovial fibroblasts in inflamed synovial tissues with RA rather than hemopoietic cells like macrophages.

**Figure 3:**
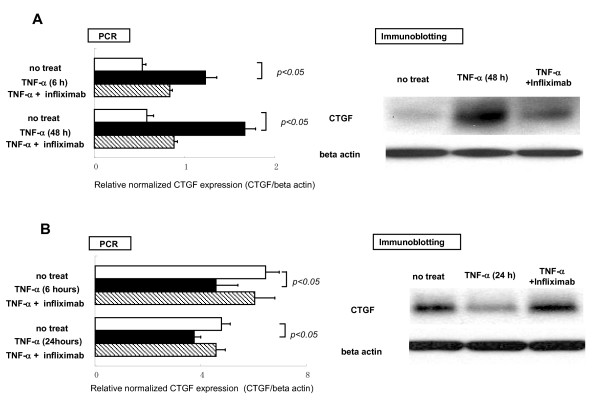
Effects of TNF-α on the regulation of connective tissue growth factor production in articular cells. CTGF production in the human synovial fibroblasts cell line (MH7A) (Figure 3A) in human chondrocytes cell line (OUMS-27) (Figure 3B) stimulated with/without TNF-α were evaluated by immunoblotting and quantitative real time PCR. TNF-α promoted CTGF production in MH7A and oppositely inhibited the production in OUMS-27.

In order to investigate how TNF-α regulates the production of CTGF in synovial fibroblasts, CTGF expression was measured in MH7A cells (human synovial fibroblasts cell line) stimulated with or without recombinant TNF-α. Furthermore, as it is well known that cartilage tissue, which is also a typical site damaged by RA, is affected by TNF-α, a similar experiment was performed using OUMS-27 cells (human condrocytes cell line). The results obtained by immunoblotting and quantitative real time PCR revealed that TNF-α enhanced the expression of CTGF in MH7A cells and this enhancement was neutralized by infliximab (Figure 3A). The influence of TNF-α on CTGF production for chondrocytes was also investigated using the OUMS-27. In contrast to synovial fibroblasts, CTGF production was oppositely diminished by TNF-α stimulation and this inhibitory effect was restored by infliximab (Figure 3B). These data suggest that TNF-α can distinctly regulate CTGF production in a cell dependent manner.

#### Synergistic effects of CTGF on osteoclastgenesis mediated with M-CSF/RANKL

**Figure 4:**
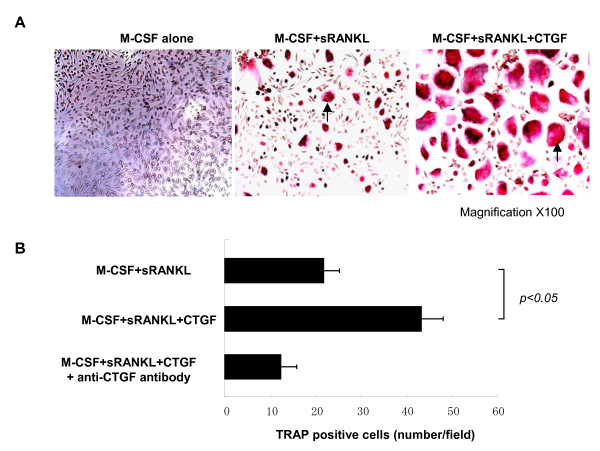
Synergistic effects of connective tissue growth factor for M-CSF/sRANKL-mediated osteoclastogenesis. Figure 4A shows pictures of TRAP staining and Figure 4B shows the numbers of TRAP positive cells. The TRAP positive cells stained by red were indicated by arrows. CTGF increased not only number but also morphological size of differentiated osteoclasts in combination with M-CSF/RANKL. Bars in Figure 4B indicate the SD.

**Figure 5:**
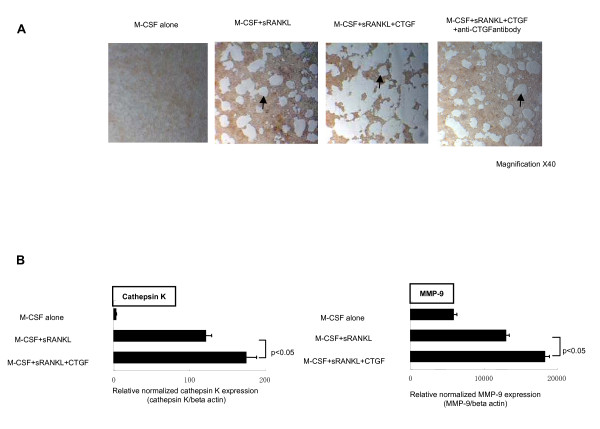
Synergistic effects of connective tissue growth factor on M-CSF/sRANKL-mediated osteoclastic function. Figure 5A shows the results of the resorption of osteoclasts on calcium phosphate. Vacant regions indicated by arrows represent the areas where the osteoclasts actually absorbed. There was no vacant region in negative control cells (M-CSF alone). In contrast to negative control, significant vacant regions were observed in osteoclasts induced by M-CSF/sRANKL. CTGF further expanded the vacant areas in combination with M-CSF/sRANKL and anti-CTGF antibody neutralized this effect. Figure 5B shows the levels of expression of osteoclasts specific proteases (MMP-9 and cathepsin-K) measured by quantitative real time RT-PCR. Synergistic effect of CTGF was also observed for M-CSF/sRANKL-mediated osteoclastogenesis. Bars in Figure 5B indicate the SD.

Next, the effect of CTGF on osteoclastogenesis was examined in order to investigate functional roles of CTGF on the RA-related bone destruction. Although CTGF alone had no capability for the differentiation of osteoclasts (data not shown), M-CSF/RANKL-mediated osteoclastogenesis was enhanced by the presence of CTGF not only in the morphological size but also in the number of osteoclasts and this enhancing effect was neutralized by anti-CTGF antibody (Figure 4A and 4B). Next, the effect of CTGF for the osteoclastic function was also investigated. Bone resorption mediated by osteoclasts, which represents vacant areas indicated by arrows in Figure 5A, was enhanced in the presence of CTGF and this effect was also neutralized by anti-CTGF antibody (Figure 5A). Furthermore, the productions of cathepsin-K and MMP-9, which are representative osteoclastic specific catalytic enzymes, were also enhanced by the presence of CTGF with M-CSF/RANKL in comparison to the absence of CTGF in the culture (Figure 5B). These data suggest CTGF promote osteoclastogenesis in the presence of M-CSF/RANKL and excessive CTGF is an important factor of aberrant osteoclasts activation in RA pathogenesis.

### CTGF activates focal adhesion kinase (FAK) and extracellular signal-regulated kinase (ERK) 1/2 through integrin αVβ3 on the osteoclasts

**Figure 6:**
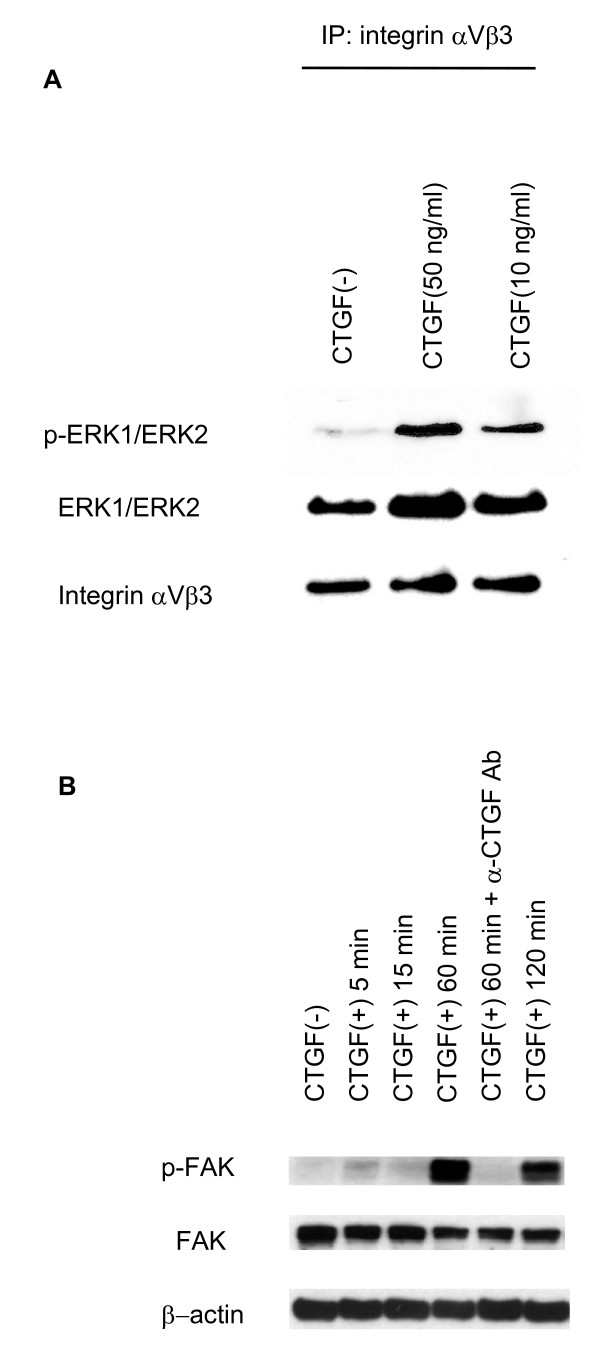
Connective tissue growth factor mediate ERK1/2 and focal adhesion kinase activation through integrin αVβ3 signal transduction. Figure 6A shows the immnoprecipitation and immunoblotting analysis. The cell extracts of osteoclasts stimulated with recombinant CTGF (10 or 50 ng/ml) at 60 minutes were precipitated using anti-integrin αVβ3 antibody and subsequently blotted with anti- phosphorylated ERK1/ERK2, conventional ERK1/ERK2, and integrin αVβ3 antibodies respectively. The phosphorylated ERK1/ERK2 was recruited with integrin αVβ3 by CTGF stimulation. Figure 6B shows the immunoblotting analysis using anti- phosphorylated FAK, conventional FAK, and β-actin antibodies in the osteoclasts extracts treated with CTGF (10 ng/ml) at 5, 15, 60, and 120 minutes in the presence or absence of anti-CTGF antibody (1 μg/ml). CTGF stimulation resulted in phosphorylation of FAK from 60 minutes and this effect was neutralized by anti-CTGF antibody suggesting activation of signal transduction pathways through integrin αVβ3.

Although a specific receptor of CTGF has not been fully identified so far, several molecules have been reported as CTGF receptors. Chen and co-workers reported that a neutralizing antibody against integrin αVβ3 significantly attenuated CTGF-mediated ERK1/2 activation and cellular migration in human breast cancer cells, indicating that the integrin αVβ3-ERK1/2 signaling pathway is crucial in mediating CTGF function [21]. Furthermore, Tan and co-workers very recently found that CTGF stimulation increased the phosphorylation of FAK and ERK via integrin αVβ3 resulting in the migration and expression of matrix metalloproteinase (MMP)-13 in human chondrosarcoma cells [22]. To date, several cell lines of evidence have shown that, among various integrins, osteoclasts express very high levels of integrin αVβ3 and it is now well accepted that this integrin is a central molecule for osteoclastic bone resorption [23]. Therefore we considered that excessive CTGF produced by synovial fibroblasts in RA contribute to increased osteoclastic function through integrin αVβ3 signaling as well as other type of cells. To assess molecular actions of CTGF on osteoclasts, immunoprecipitation and immunoblotting analysis were performed. Figure 6 indicated that phosphorylated ERK1/2 was recruited by integrin αVβ3 upon CTGF stimulation (Figure 6A), and CTGF also induced FAK phosporylation (Figure 6B). These data suggest that integrin αVβ3 is a receptor of CTGF and CTGF could enhance osteoclastic function through activation of integrin αVβ3 signaling transduction pathways such as ERK1/2 and FAK phosphorylation.

## Discussion

**Figure 7:**
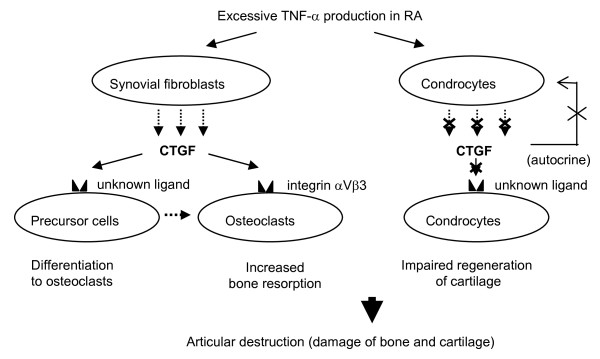
Hypothesis of the role of connective tissue growth factor in the possible rheumatoid arthritis pathogenesis.

This study was conducted to investigate roles of CTGF for the possible pathogenesis of RA. We found novel findings as follows: **I**) serum levels of CTGF in RA were significantly greater than those of disease controls (SLE and SS) and normal healthy controls (Figure 1A); **II**) serum concentrations of CTGF were significantly elevated in patients with active RA compared to inactive RA (Figure 1B), furthermore, a significant reduction of the serum CTGF level was observed by infliximab administration concomitant with the disease amelioration (Figure 1C); **III**) immunohistochemical studies reveal that CTGF appears to be massively produced by synovial fibloblasts in RA (Figure 2); **IV**) the production of CTGF in synovial fibroblasts was up-regulated by TNF-α stimulation (Figure 3A) and those of CTGF in chondrocytes were oppositely down-regulated (Figure 3B); **V**) CTGF possessed a synergistic effect in combination with MCSF/RANKL for osteoclastogenesis through integrin αVβ3 signaling on the osteoclasts (Figures 4, 5 and 6). Taken together, a schematic hypothesis of the role of CTGF in the RA pathogenesis is presented in Figure 7. It has been reported that CTGF adenovirus vector transfection into knee joints of mice induces linier overexpression of CTGF in the synovium and results in cartilage damages with increasing of mRNA cording for degradative enzymes such as MMP-3 [24]. Manns and co-workers also reported that CTGF is up-regulated in an experimental animal model of RA, and they have shown that treatment with thrombospondin (TSP)-1-derived peptide is associated with down-regulation of CTGF concomitant with the disease amelioration [25]. Furthermore, the null knock-down of the CTGF gene dramatically inhibits osteoclast-like formation in mice [26]. Their reports indicate that CTGF has a significant role for RA pathogenesis and our present data can support these previous reports.

The synovial tissue in the inflamed joints of RA can invade bones and this is supported by the invasive nature of the synovial fibroblasts gaining the capacity to move and penetrate into cartilages and bones. Osteoclasts are the sole bone-absorbing cells and the functions of osteoclasts are increased in RA patients as well as in patients with osteoporosis. Osteoclasts are commonly found within the erosive pit at the interference of synovial inflammatory tissue and subchondral bone [27]. RANKL is a membrane-residing protein on osteoblasts and recognizes its receptor (RANK) expressed on marrow macrophages, promoting them to differentiate into the osteoclast phenotype in the presence of M-CSF [28-30]. These molecules are expressed locally in the synovial tissues of RA patients and TNF-α is an inducer of RANKL expression as well as IL-1 and IL-6 [31,32]. Therefore, aberrant osteoclastogenesis play an important role in the development of RA and this process is further positively regulated by proinflammatory cytokines like TNF-α under pathogenic conditions. As shown in Figure 2, strong CTGF expression appeared to be observed at inflamed synovial fibroblasts derived from surgical samples of RA patients. Furthermore, CTGF was upregulated by TNF-α in human synovial fibroblasts cell line, MH7A, as shown in Figure 3A. The present data indicate that excessive CTGF production from synovial fibroblasts with RA patients induced by TNF-α can promote aberrant activation of osteoclasts in combination with RANKL/M-CSF, resulting in bone destruction.

In addition, we found a CTGF receptor, integrin αVβ3, on the osteoclasts. Integrins are heterodimec adhesion receptors that mediate cell-matrix interaction. Osteoclasts exhibit high expression of the integrin αVβ3, which binds to a variety of extracellular matrix proteins including vitronectin, osteopontin, and bone sialoprotein. Arg-Gly-Asp (RGD)-containing peptides, RGD-mimitics, and blocking antibodies to integrin αVβ3 were shown to inhibit bone resorption in vitro and in vivo, suggesting that this integrin may play an important role in osteoclast function [23]. It has been shown that CTGF could interact with integrin αVβ3 in other types of cells, however, it has not been shown to our knowledge that integrin αVβ3 on the osteoclasts interact with CTGF. Furthermore, blocking of the integrin αVβ3 signaling pathway has been shown to increase bone mineral density in women with postmenoposal osteoporosis [20]. CTGF/Integrin αVβ3 pathway is now receiving considerable attention as a therapeutic target in diseases associated with increased bone resorption, such as RA. Therefore, we insist that our findings are very important for the understanding of CTGF/integrin αVβ3 contribution to disease progression in RA.

Recently anti-TNF-α blocking reagents are becoming more widely available in practical treatments for RA patients [33]. Significant beneficial effects of infliximab on bone destruction have been reported even in RA patients without improvements of RA-related clinical symptoms [34]. Therefore, other mechanisms besides the blocking of inflammatory reactions like CRP or ESR elevation appear to be associated with infliximab-mediated inhibition of articular damage in RA patients. The inhibition of CTGF production from synovial cells mediated by infliximab may play an important role in the blocking of bone destruction in RA patients. In this study, we also measured serum levels of TNF-α concomitant with CTGF, however, significant correlation with serum levels of TNF-α and CTGF was not observed (data not shown). These data suggest that high levels of serum CTGF in active RA patients are not merely mediated by the production from synovial fibroblasts with TNF-α stimulation. Furthermore, although the production of CTGF has been reported to be induced by TGF-α in connective tissues [14], no specific correlation was also observed between CTGF and TGF-α concentrations in the sera of RA patients (data not shown). One possible explanation for these dissociations, high levels of serum CTGF production in active RA is mediated by other multiple stimulating factors or productive sites. In fact, IL-6, which is also the main proinflamatory cytokine related to RA pathogenesis, was reported as the stimulating factor of CTGF production [35]. Furthermore, vascular endothelial cells, which are also affected by RA, are known as major productive sites of CTGF [14]. These factors might be involved in mechanisms of the elevation of serum CTGF in active RA.

In our study, TNF-α can induce CTGF production from synovial cells. In contrast, TNF-α could oppositely inhibit the production of CTGF from chondrocytes. It has been proposed that CTGF contributes to maintaining cartilage homeostasis by the autocrine system [14]. CTGF also might promote the direct proliferation of osteoblasts [14]. Therefore, CTGF may possess positive regulatory functions for chondrocytes and osteoblasts to keep physiological articular homeostasis. Therefore, reduction of CTGF from condrocytes may result in cartilage damage. These dual mechanisms of CTGF appear to be important for the pathogenesis of RA. It was reported that treatment with TNF-α significantly increased CTGF on cultured mesangial cells and pancreatic stellate cells [36,37]. On the contrary, Yu and co-workers recently reported that TNF-α suppressed TGF-α mediated CTGF production by alteration of TGF-α signal transduction [38]. Although the precise mechanism for why CTGF production can be distinctively regulated by TNF-α has not been elucidated to date, one possible explanation is that TNF-α has multiple biological functions depending upon cell types, and multiple receptors (TNF-receptor 1 and TNF-receptor 2) are responsible for the appearance of the functions. Wehling and co-workers recently reported that IL-1β and TNF-α inhibited chondrogenesis from mesenchymal stem cells into chondrocytes in a dose-dependent manner and this was associated with a marked activation of NF-κB [39], also Polzer and co-workers reported that although wild-type mice showed no signs of cartilage damage, human TNF transgenic mice exhibited progressive proteoglycan loss starting at the clinical onset of arthritis [40]. These data suggest chondrocytes and cartilage tissue were given destructive effects by TNF-α. Otherwise, Mun and co-workers reported that TNF-α-induced interleukin-32, which is a recently discovered proinflamatory cytokine that appears to play a critical role in human rheumatoid arthritis (RA), is positively regulated via the Syk/protein kinase Cδ/JNK pathway in rheumatoid synovial fibroblasts [41]. Gao also reported that the proinflammatory cytokines IL-1β and TNF-α induce the expression of Synoviolin, an E3 ubiquitin ligase, in mouse synovial fibroblasts via the Erk1/2-ETS1 pathway indicating that the proinflammatory cytokines IL-1β and TNF-α induce the overgrowth of synovial cells by upregulating Synoviolin expression via the Erk1/-ETS1 pathway [42]. Their reports indicate that TNF-α appears to possess proinflamatory or proliferative effects against synovial fibroblasts resulting in further diseases progression of RA. In the diseases condition with RA, TNF-α mediates various biological effects depend on the cell types in order to progress or maintain the disease effectively. This may be an important mechanism of efficacy in TNF-α blocking therapy, which can suppress multiple functions of TNF-α, for the inhibition of bone destruction and/or promotion of cartilage regeneration in patients with RA.

Our previous report of mass spectrometric analysis of low molecular weight serum proteins of RA patients shows that CTGF was more frequently detected in the low molecular weight fraction after infliximab treatment of RA patients [10]. The precise reasons for this discrepancy are still unclear. However, the apparent molecular mass of the serum CTGF molecule or molecular complex(es) in RA patients may be out of the range of the ultrafiltration pre-fractionation used in previous protein analysis. Furthermore, the molecular size of the recovered proteins may be changed by partial proteolysis or dissociation of protein complex(es) of CTGF after infliximab treatment.

Taken together, our study indicated an important role of CTGF in the development of bone destruction in patients with RA and suggested a mechanism explaining the efficacy of anti-TNF-α antibodies in the prevention of bone destruction in RA. The present data suggest that CTGF plays significant roles in the pathogenesis of RA especially through aberrant activation of osteoclasts and disturbance of cartilage tissue homeostasis, thus resulting in articular destruction. In addition, it is possible that the blockade of the CTGF/integrin αVβ3 signaling pathway by neutralizing antibody has beneficial effects in the treatment of RA. The administration of anti-CTGF antibodies to RA model mice *in vivo *will be conducted in the future. These results may open new therapeutic strategies for patients with RA and the possibility of development of more specific biological therapies rather than antibodies to TNF-α.

## Conclusions

This study was conducted to investigate roles of CTGF in the possible pathogenesis of RA. Our data indicate that excessive CTGF induced by TNF-α can promote aberrant activation of osteoclasts in combination with RANKL/M-CSF, resulting in bone destruction. In contrast, TNF-α could oppositely inhibit the production of CTGF from chondrocytes. It has been proposed that CTGF contributes to maintaining cartilage homeostasis by the autocrine system. Therefore, reduction of CTGF from condrocytes may result in cartilage damage. These dual mechanisms of CTGF appear to be important in the pathogenesis of RA. This may be an important mechanism of efficacy in TNF-α blocking reagents therapy on the inhibition of bone destruction and/or promotion of cartilage regeneration in patients with RA.

## Abbreviations

CRP: C reactive protein; CTGF: connective tissue growth factor; ERK: extracellular signal-regulated kinase; ESR: erythrocyte sedimentation rate; FAK: focal adhesion kinase; M-CSF: Macrophage colony-stimulating factor; MMP: matrix metalloproteinase; NF-κB: nuclear factor-kappa B; OA: osteoarthropathy; RA: rheumatoid arthritis; SLE: systemic lupus erhythematosus; SS: Sjögren's Syndrome; TRAP: tartrate-resistant acid phosphatase; WBC: white blood cell.

## Competing interests

The authors declare that they have no competing interests.

## Authors' contributions

KN and MF mainly carried out the experiments and equally contributed to this study. IS conducted this study in its entirety.

## References

[B1] Feldmann M Brennan FM Maini RN Role of cytokines in rheumatoid arthritis Annu Rev Immunol 1996 14 397 440 10.1146/annurev.immunol.14.1.397 8717520

[B2] Fassbender HG Seibel M Hebert T Pathways of destruction in metacarpal and metatarsal joints of patients with rheumatoid arthritis Scand J Rheumatol 1992 21 10 16 10.3109/03009749209095055 1570480

[B3] Raisz LG Pathogenesis of osteoporosis: concepts, conflicts, and prospects J Clin Invest 2005 115 3318 3325 10.1172/JCI27071 16322775 PMC1297264

[B4] Teitelbaum SL Osteoclasts: what do they do and how do they do it? Am J Pathol 2007 170 427 435 10.2353/ajpath.2007.060834 17255310 PMC1851862

[B5] Feldmann M Maini RN Anti-TNF alpha therapy of rheumatoid arthritis: what have we learned? Annu Rev Immunol 2001 19 163 196 10.1146/annurev.immunol.19.1.163 11244034

[B6] Klimiuk PA Sierakowski S Domyslawska I Chwiecko J Regulation of serum chemokines following infliximab therapy in patients with rheumatoid arthritis Clin Exp Rheumatol 2006 24 529 533 17181921

[B7] Maini RN Elliott MJ Brennan FM Williams RO Chu CQ Paleolog E Charles PJ Taylor PC Feldmann M Monoclonal anti-TNF alpha antibody as a probe of pathogenesis and therapy of rheumatoid disease Immunol Rev 1995 144 195 223 10.1111/j.1600-065X.1995.tb00070.x 7590814

[B8] Redlich K Hayer S Ricci R David JP Tohidast-Akrad M Kollias G Steiner G Smolen JS Wagner EF Schett G Osteoclasts are essential for TNF-alpha-mediated joint destruction J Clin Invest 2002 110 1419 1427 12438440 10.1172/JCI15582 PMC151809

[B9] Maini R St Clair EW Breedveld F Furst D Kalden J Weisman M Smolen J Emery P Harriman G Feldmann M Lipsky P Infliximab (chimeric anti-tumour necrosis factor alpha monoclonal antibody) versus placebo in rheumatoid arthritis patients receiving concomitant methotrexate: a randomised phase III trial. ATTRACT Study Group Lancet 1999 354 1932 1939 10.1016/S0140-6736(99)05246-0 10622295

[B10] Sekigawa I Yanagida M Iwabuchi K Kaneda K Kaneko H Takasaki Y Jung G Sone S Tanaka Y Ogawa H Takamori K Protein biomarker analysis by mass spectrometry in patients with rheumatoid arthritis receiving anti-tumor necrosis factor-alpha antibody therapy Clin Exp Rheumatol 2008 26 261 267 18565247

[B11] Bradham DM Igarashi A Potter RL Grotendorst GR Connective tissue growth factor: a cysteine-rich mitogen secreted by human vascular endothelial cells is related to the SRC-induced immediate early gene product CEF-10 J Cell Biol 1991 114 1285 1294 10.1083/jcb.114.6.1285 1654338 PMC2289134

[B12] Grotendorst GR Connective tissue growth factor: a mediator of TGF-beta action on fibroblasts Cytokine Growth Factor Rev 1997 8 171 179 10.1016/S1359-6101(97)00010-5 9462483

[B13] Perbal B CCN proteins: multifunctional signalling regulators Lancet 2004 363 62 64 10.1016/S0140-6736(03)15172-0 14723997

[B14] Takigawa M Nakanishi T Kubota S Nishida T Role of CTGF/HCS24/ecogenin in skeletal growth control J Cell Physiol 2003 194 256 266 10.1002/jcp.10206 12548546

[B15] Arnett FC Edworthy SM Bloch DA McShane DJ Fries JF Cooper NS Healey LA Kaplan SR Liang MH Luthra HS The American Rheumatism Association 1987 revised criteria for the classification of rheumatoid arthritis Arthritis Rheum 1988 31 315 324 10.1002/art.1780310302 3358796

[B16] Vitali C Bombardieri S Jonsson R Moutsopoulos HM Alexander EL Carsons SE Daniels TE Fox PC Fox RI Kassan SS Pillemer SR Talal N Weisman MH European Study Group on Classification Criteria for Sjögren's Syndrome Classification criteria for Sjogren's syndrome: a revised version of the European criteria proposed by the American-European Consensus Group Ann Rheum Dis 2002 61 554 558 10.1136/ard.61.6.554 12006334 PMC1754137

[B17] Itoh Y Hayashi H Miyazawa K Kojima S Akahoshi T Onozaki K 17beta-estradiol induces IL-1alpha gene expression in rheumatoid fibroblast-like synovial cells through estrogen receptor alpha (ERalpha) and augmentation of transcriptional activity of Sp1 by dissociating histone deacetylase 2 from ERalpha J Immunol 2007 178 3059 3066 17312152 10.4049/jimmunol.178.5.3059

[B18] Shinoda Y Ogata N Higashikawa A Manabe I Shindo T Yamada T Kugimiya F Ikeda T Kawamura N Kawasaki Y Tsushima K Takeda N Nagai R Hoshi K Nakamura K Chung UI Kawaguchi H Kruppel-like factor 5 causes cartilage degradation through transactivation of matrix metalloproteinase 9 J Biol Chem 2008 283 24682 24689 10.1074/jbc.M709857200 18617520 PMC3259811

[B19] Nakao K Kubota S Doi H Eguchi T Oka M Fujisawa T Nishida T Takigawa M Collaborative action of M-CSF and CTGF/CCN2 in articular chondrocytes: possible regenerative roles in articular cartilage metabolism Bone 2005 36 884 892 10.1016/j.bone.2004.10.015 15820145

[B20] Murphy MG Cerchio K Stoch SA Gottesdiener K Wu M Recker R Effect of L-00084 an alphaVbeta3 integrin antagonist, on markers of bone turnover and bone mineral density in postmenopausal osteoporotic women J Clin Endocrinol Metab 2005 90 2022 2028 10.1210/jc.2004-2126 15687321

[B21] Chen PS Wang MY Wu SN Su JL Hong CC Chuang SE Chen MW Hua KT Wu YL Cha ST Babu MS Chen CN Lee PH Chang KJ Kuo ML CTGF enhances the motility of breast cancer cells via an integrin-alphavbeta3-ERK1/2-dependent S100A4-upregulated pathway J Cell Sci 2007 120 2053 2065 10.1242/jcs.03460 17550972

[B22] Tan TW Lai CH Huang CY Yang WH Chen HT Hsu HC Fong YC Tang CH CTGF enhances migration and MMP-13 up-regulation via alphavbeta3 integrin, FAK, ERK, and NF-kappaB-dependent pathway in human chondrosarcoma cells J Cell Biochem 2009 107 345 356 10.1002/jcb.22132 19301259

[B23] Nakamura I Duong le T Rodan SB Rodan GA Involvement of alpha(v)beta3 integrins in osteoclast function J Bone Miner Metab 2007 25 337 344 10.1007/s00774-007-0773-9 17968485

[B24] Blaney Davidson EN Vitters EL Mooren FM Oliver N Berg WB Kraan PM van der Connective tissue growth factor/CCN2 overexpression in mouse synovial lining results in transient fibrosis and cartilage damage Arthritis Rheum 2006 54 1653 1661 10.1002/art.21795 16646035

[B25] Manns JM Uknis AB Rico MC Agelan A Castaneda J Arango I Barbe MF Safadi FF Popoff SN DeLa Cadena RA A peptide from thrombospondin 1 modulates experimental erosive arthritis by regulating connective tissue growth factor Arthritis Rheum 2006 54 2415 2422 10.1002/art.22021 16869004

[B26] Shimo T Kubota S Yoshioka N Ibaragi S Isowa S Eguchi T Sasaki A Takigawa M Pathogenic role of connective tissue growth factor (CTGF/CCN2) in osteolytic metastasis of breast cancer J Bone Miner Res 2006 21 1045 1059 10.1359/jbmr.060416 16813525

[B27] Schett G Rheumatoid arthritis: inflammation and bone loss Wien Med Wochenschr 2006 156 34 41 10.1007/s10354-005-0244-7 16465612

[B28] Gravallese EM Manning C Tsay A Naito A Pan C Amento E Goldring SR Synovial tissue in rheumatoid arthritis is a source of osteoclast differentiation factor Arthritis Rheum 2000 43 250 258 10.1002/1529-0131(200002)43:2<250::AID-ANR3>3.0.CO;2-P 10693863

[B29] Shigeyama Y Pap T Kunzler P Simmen BR Gay RE Gay S Expression of osteoclast differentiation factor in rheumatoid arthritis Arthritis Rheum 2000 43 2523 2530 10.1002/1529-0131(200011)43:11<2523::AID-ANR20>3.0.CO;2-Z 11083276

[B30] Sato K Suematsu A Okamoto K Yamaguchi A Morishita Y Kadono Y Tanaka S Kodama T Akira S Iwakura Y Cua DJ Takayanagi H Th17 functions as an osteoclastogenic helper T cell subset that links T cell activation and bone destruction J Exp Med 2006 203 2673 2682 10.1084/jem.20061775 17088434 PMC2118166

[B31] Lam J Takeshita S Barker JE Kanagawa O Ross FP Teitelbaum SL TNF-alpha induces osteoclastogenesis by direct stimulation of macrophages exposed to permissive levels of RANK ligand J Clin Invest 2000 106 1481 1488 10.1172/JCI11176 11120755 PMC387259

[B32] Wei S Kitaura H Zhou P Ross FP Teitelbaum SL IL-1 mediates TNF-induced osteoclastogenesis J Clin Invest 2005 115 282 290 15668736 10.1172/JCI23394 PMC544608

[B33] Lipsky PE Heijde DM van der St Clair EW Furst DE Breedveld FC Kalden JR Smolen JS Weisman M Emery P Feldmann M Harriman GR Maini RN Anti-Tumor Necrosis Factor Trial in Rheumatoid Arthritis with Concomitant Therapy Study Group Infliximab and methotrexate in the treatment of rheumatoid arthritis. Anti-Tumor Necrosis Factor Trial in Rheumatoid Arthritis with Concomitant Therapy Study Group N Engl J Med 2000 343 1594 1602 10.1056/NEJM200011303432202 11096166

[B34] Smolen JS Han C Bala M Maini RN Kalden JR Heijde D van der Breedveld FC Furst DE Lipsky PE Evidence of radiographic benefit of treatment with infliximab plus methotrexate in rheumatoid arthritis patients who had no clinical improvement: a detailed subanalysis of data from the anti-tumor necrosis factor trial in rheumatoid arthritis with concomitant therapy study Arthritis Rheum 2005 52 1020 1030 10.1002/art.20982 15818697

[B35] Leung JC Chan LY Tam KY Tang SC Lam MF Cheng AS Chu KM Lai KN Regulation of CCN2/CTGF and related cytokines in cultured peritoneal cells under conditions simulating peritoneal dialysis Nephrol Dial Transplant 2009 24 458 469 10.1093/ndt/gfn524 18805993

[B36] Cooker LA Peterson D Rambow J Riser ML Riser RE Najmabadi F Brigstock D Riser BL TNF-alpha, but not IFN-gamma, regulates CCN2 (CTGF), collagen type I, and proliferation in mesangial cells: possible roles in the progression of renal fibrosis Am J Physiol Renal Physiol 2007 293 F157 165 10.1152/ajprenal.00508.2006 17376761

[B37] Karger A Fitzner B Brock P Sparmann G Emmrich J Liebe S Jaster R Molecular insights into connective tissue growth factor action in rat pancreatic stellate cells Cell Signal 2008 20 1865 1872 10.1016/j.cellsig.2008.06.016 18639630

[B38] Yu F Chou CW Chen CC TNF-alpha suppressed TGF-beta-induced CTGF expression by switching the binding preference of p300 from Smad4 to p65 Cell Signal 2009 21 867 872 10.1016/j.cellsig.2009.01.030 19385047

[B39] Wehling N Palmer GD Pilapil C Liu F Wells JW Muller PE Evans CH Porter RM Interleukin-1beta and tumor necrosis factor alpha inhibit chondrogenesis by human mesenchymal stem cells through NF-kappaB-dependent pathways Arthritis Rheum 2009 60 801 812 10.1002/art.24352 19248089 PMC2688727

[B40] Polzer K Schett G Zwerina J The lonely death: chondrocyte apoptosis in TNF-induced arthritis Autoimmunity 2007 40 333 336 10.1080/08916930701356721 17516222

[B41] Mun SH Kim JW Nah SS Ko NY Lee JH Kim JD Kim do K Kim HS Choi JD Kim SH Lee CK Park SH Kim BK Kim HS Kim YM Choi WS Tumor necrosis factor alpha-induced interleukin-32 is positively regulated via the Syk/protein kinase Cdelta/JNK pathway in rheumatoid synovial fibroblasts Arthritis Rheum 2009 60 678 685 10.1002/art.24299 19248119

[B42] Gao B Calhoun K Fang D The proinflammatory cytokines IL-1beta and TNF-alpha induce the expression of Synoviolin, an E3 ubiquitin ligase, in mouse synovial fibroblasts via the Erk1/2-ETS1 pathway Arthritis Res Ther 2006 8 R172 10.1186/ar2081 17105652 PMC1794516

